# GENOMIC ANALYSIS OF HUMAN ISOGENIC APOE IPSC-DERIVED INHIBITORY GABAERGIC NEURONS

**DOI:** 10.1093/geroni/igz038.2315

**Published:** 2019-11-08

**Authors:** Lisa Ellerby, Sicheng Song, Sean Mooney, Stephen Scheeler, Swati Naphade

**Affiliations:** 1 The Buck Institute, Novato, California, United States; 2 University of Washington, Seattle, Washington, United States; 3 Buck Institute for Research on Aging, Novato, California, United States

## Abstract

Isoforms of ApoE modify the risk for Alzheimer’s disease (AD), cardiovascular disease and are also associated with exceptional longevity. Specifically, the ApoE E2 allele is associated with lower risk of AD-related neurodegeneration and with exceptional longevity, while the E4 allele is a major risk factor for AD and is associated with higher levels of Abeta deposition in the brain. The mechanisms modulating extended lifespan/healthspan mediated by E2 compared to E3 and E4 genotypes are not clear. One hypothesis is that the E2 allele is neuroprotective and compensates for neuronal dysfunction induced by misfolded protein expression in aging and disease. To understand the molecular basis of the protective effect of the E2 allele we performed transcriptomic analysis of isogenic iPSCs with E2E2 and E4E4 genotypes differentiated into inhibitory GABAergic neurons. Our analysis revealed that ApoE2 inhibitory GABAergic neurons regulate genes involved in nuclear division, DNA integrity and DNA damage checkpoint.

